# Overall Survival After Treatment Failure Among Patients With Rectal Cancer

**DOI:** 10.1001/jamanetworkopen.2023.40256

**Published:** 2023-10-30

**Authors:** Markus Diefenhardt, Daniel Martin, Maximilian Fleischmann, Ralf-Dieter Hofheinz, Michael Ghadimi, Claus Rödel, Emmanouil Fokas

**Affiliations:** 1 Department of Radiotherapy and Oncology, University Hospital, Goethe University Frankfurt, Frankfurt, Germany; 2 Frankfurt Cancer Institute, Goethe University Frankfurt, Frankfurt, Germany; 3 German Cancer Research Center and German Cancer Consortium, partner site Frankfurt, Frankfurt, Germany; 4 Department of Medical Oncology, University Hospital Mannheim, University Heidelberg, Heidelberg, Germany; 5 Department of General, Visceral, and Paediatric Surgery, University Medical Center Göttingen, University Göttingen, Göttingen, Germany

## Abstract

**Importance:**

Oncologic outcomes among patients with rectal cancer after developing local recurrence and/or distant metastases remain poorly studied.

**Objective:**

To analyze the trend of overall survival after treatment failure for patients with rectal cancer within three consecutive phase 2 or 3 trials of the German Rectal Cancer Study Group.

**Design, Setting, and Participants:**

This cohort study is a post hoc analysis of 3 randomized phase 2 or 3 trials (CAO/ARO/AIO-94, -04, and -12 trials, conducted in Germany) that included 1948 patients with locally advanced rectal adenocarcinoma. The CAO/ARO/AIO-94 trial recruited patients between February 1995 and September 2002, the CAO/ARO/AIO-04 trial recruited patients between July 2006 and February 2010, and the CAO/ARO/AIO-12 trial recruited patients between June 2015 and January 2018. Statistical analysis was conducted between September 2022 and March 2023.

**Exposures:**

A total of 119 of 391 patients in the CAO/ARO/AIO-94 trial group A, 295 of 1236 patients in the CAO/ARO/AIO-04 trial, and 69 of 306 in the CAO/ARO/AIO-12 trial experienced treatment failure (R2 resection or local recurrence or distant metastases) and were included in further analyses.

**Main Outcomes and Measures:**

Characteristics of treatment failure and overall survival were assessed in all 3 trial cohorts.

**Results:**

Of the 1948 patients treated in the 3 trials, 15 were excluded because of missing data. Of the remaining 1933 patients (median age, 62.5 years [range, 19-84 years]; 1363 men [71%] and 570 women [29%]) with locally advanced rectal adenocarcinoma (cT3 or 4 or cN+) treated within 3 consecutive clinical trials, 483 experienced treatment failure and were analyzed. After a median follow-up of 36 months (IQR, 24-51 months) for all patients, overall survival after treatment failure was significantly improved in the CAO/ARO/AIO-04 trial (at 3 years, 44% [IQR, 37%-51%]; hazard ratio [HR], 0.61 [95% CI, 0.47-0.79]) and further improved in the CAO/ARO/AIO-12 trial (at 3 years, 73% [IQR, 60%-87%]; HR, 0.32 [95% CI, 0.18-0.54]) compared with the CAO/ARO/AIO-94 trial (at 3 years, 30% [IQR, 22%-39%]) (both *P* < .001). Distant metastasis was the main reason for treatment failure throughout a 5-year follow-up (range, 67%-87%), and the relative risk for treatment failure was highest in the first 18 months in all 3 trials. ypTNM stage was significantly associated with the risk and time interval to treatment failure. Improvement in overall survival after treatment failure was independent of sex.

**Conclusions and Relevance:**

This cohort study suggests that advancements in salvage strategies during the past decades have likely improved overall survival among patients with rectal cancer who experienced treatment failure.

## Key Points

**Question:**

Has overall survival after treatment failure improved for patients with rectal cancer after neoadjuvant chemoradiotherapy or total neoadjuvant treatment and total mesorectal excision surgery over the past decades?

**Findings:**

In this cohort study of 1948 patients treated with neoadjuvant fluorouracil-based chemoradiotherapy and adjuvant chemotherapy with or without oxaliplatin or with total neoadjuvant treatment, 483 patients experienced treatment failure. Overall survival after treatment failure was significantly improved over 3 generations of phase 2 or 3 trials (CAO/ARO/AIO-94, -04, and -12) by the German Rectal Cancer Study Group.

**Meaning:**

This study suggests that implemented surveillance programs and multidisciplinary approaches should further be evaluated within clinical trials to improve salvage strategies for overall survival after treatment failure for patients with rectal cancer.

## Teaser

This cohort study analyzes the trend of overall survival after treatment failure for patients with rectal cancer in 3 consecutive phase 2 or 3 trials of the German Rectal Cancer Study Group.

## Introduction

Intensification of neoadjuvant fluorouracil-based chemoradiotherapy (FU CRT) has led to the new treatment paradigm of total neoadjuvant treatment (TNT) for locally advanced rectal cancer. The RAPIDO (Rectal Cancer and Preoperative Induction Therapy Followed by Dedicated Operation)^1^ and the PRODIGE23 (Actions Concertées dans les Cancers Colorectaux et Digestifs)^2^ randomized phase 3 trials demonstrated that TNT strategies can improve disease-free survival (DFS) and enhance local tumor regression but failed to provide evidence for improved overall survival (OS) after a median follow-up of 4.6 years and 46.5 months, respectively. Despite the improvement in oncologic outcomes, 1 in 4 patients still experienced local recurrence (LR) or distant metastasis (DM).^3,4^ With the lack of randomized and limited retrospective trials, evidence for optimal surveillance strategies and salvage treatment remains unclear. We examined OS after treatment failure among patients treated within the consecutive CAO/ARO/AIO-94, CAO/ARO/AIO-04, and the CAO/ARO/AIO-12 prospective, 2-group randomized phase 2 or 3 trials^5,6,7^ of the German Rectal Cancer Study Group (GRCSG) who experienced local and/or distant treatment failure after initial CRT or TNT and total mesorectal excision with or without adjuvant chemotherapy.

## Methods

The CAO/ARO/AIO-94 trial recruited patients between February 1995 and September 2002, the CAO/ARO/AIO-04 trial recruited patients between July 2006 and February 2010, and the CAO/ARO/AIO-12 trial recruited patients between June 2015 and January 2018 (NCT00349076).^5,6,7,8,9,10^ The design and oncologic outcomes have been previously published.^5,6,7,8,9,10^ The CAO/ARO/AIO-94 trial had an extended follow-up of 10 years, whereas the CAO/ARO/AIO-04 and CAO/ARO/AIO-12 trials had a regular follow-up of 5 years.^5,6^ All sites obtained medical ethics committee approval and written patient informed consent. In the CAO/ARO/AIO-94 trial, only patients treated with neoadjuvant CRT (group A) were screened for this study.^5^ This report followed the Strengthening the Reporting of Observational Studies in Epidemiology (STROBE) reporting guideline for cohort studies.^11^

### Statistical Analysis

Statistical analysis was conducted between September 2022 and March 2023. Treatment failure events were defined as R2 resection, occurrence of LR after R0 or R1 resection, or DM, whichever occurred first. Time to treatment failure was defined as the time from randomization to the treatment failure event. The Spearman correlation was used to examine the correlation between DFS and OS, both defined from randomization. The *t* test was used to evaluate differences in time to treatment failure according to ypTNM classification. The Pearson χ^2^ test was used to analyze differences in sites for treatment failure by location (low, 0-5 cm; middle, >5-10 cm; high, >10 cm of the anal verge) or sex. The rma function in the metafor package of R, version 4.2.1, software (R Project for Statistical Computing) was used to examine sex differences for OS improvement. Median follow-up was calculated with the reverse Kaplan-Meier approach. Overall survival was examined with the log-rank test and in a Cox proportional hazards regression model. The proportional hazard assumptions were tested with the cox.zph function in the survival package of R, version 4.2.1, software and revealed no violations of the proportional hazard assumptions. All *P* values were from 2-sided tests and results were deemed statistically significant at *P* < .05.

## Results

**Figure 1.**
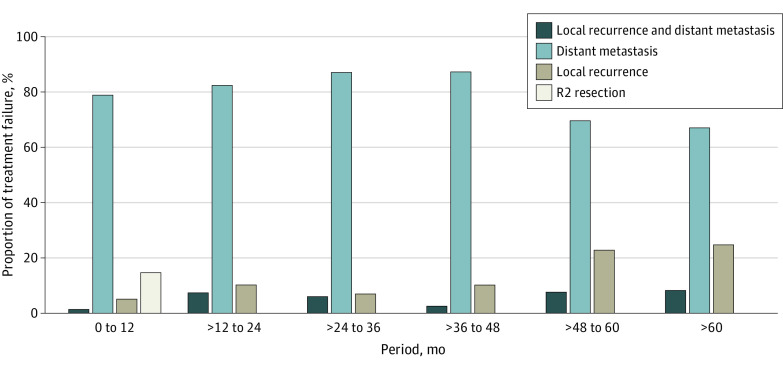
Patterns of Treatment Failure Within Certain Follow-Up Periods The proportion of treatment failure was calculated by dividing the number of treatment failures for a specific reason by all treatment failures within a certain follow-up period.

Of the 1948 patients treated in the 3 trials, 15 were excluded because of missing data. Of the remaining 1933 patients (median age, 62.5 years [range, 19-84 years]; 1363 men [71%] and 570 female [29%]) with locally advanced rectal cancer (cT3 or 4 or cN+), treatment failure occurred among 119 of 391 patients in the CAO/ARO/AIO-94 trial, 295 of 1236 patients in the CAO/ARO/AIO-04 trial, and 69 of 306 patients in the CAO/ARO/AIO-12 trial. eFigure 1 in Supplement 1 shows the flow diagram of the present analysis. The median time to treatment failure was comparable between the trials (CAO/ARO/AIO-94, 16 months [IQR, 9-34 months]; CAO/ARO/AIO-04, 14 months [IQR, 4-25 months]; CAO/ARO/AIO-12, 15 months [IQR, 10-26 months]). Distant metastasis was the main cause of treatment failure throughout a 5-year follow-up (range, 67%-87%) (Figure 1). However, we found that the relative risk of experiencing a LR among patients with treatment failure increased from 5% in the first 12 months to 23% in the fourth year of follow-up.

**Figure 2.**
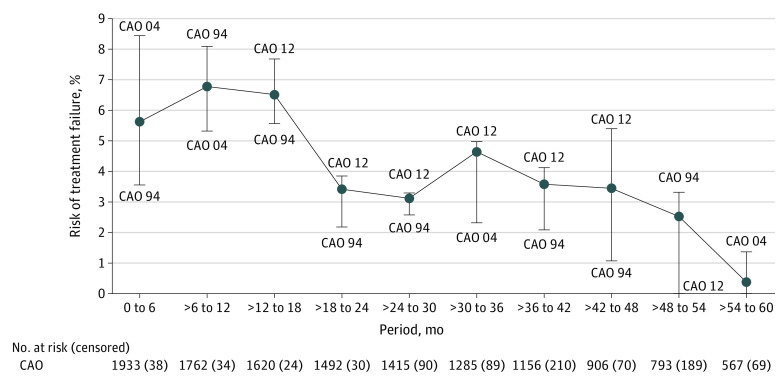
Risk of Treatment Failure Within Certain Follow-Up Periods, Depending on Trial Censored patients are patients who were lost during the specific follow-up period. The upper error bar indicates the trial with the highest incidence of treatment failure in the certain follow-up period. The middle line refers to the trial with the intermediate incidence of treatment failure. The lower error bar indicates the trial that had the lowest incidence of treatment failure. CAO 04 indicates CAO/ARO/AIO-04 trial; CAO 12, CAO/ARO/AIO-12 trial; and CAO 94, CAO/ARO/AIO-94 trial.

In all 3 trials, most treatment failures occurred within the first 18 months after randomization (Figure 2). Of note, 9% of all treatment failures (11 of 119) in the CAO/ARO/AIO-94 trial occurred after the regular 60-month follow-up period. eFigure 2A to D in Supplement 1 provides additional plots of the risk of treatment failure within specific time periods in the overall cohort and separately for each trial.

**Figure 3.**
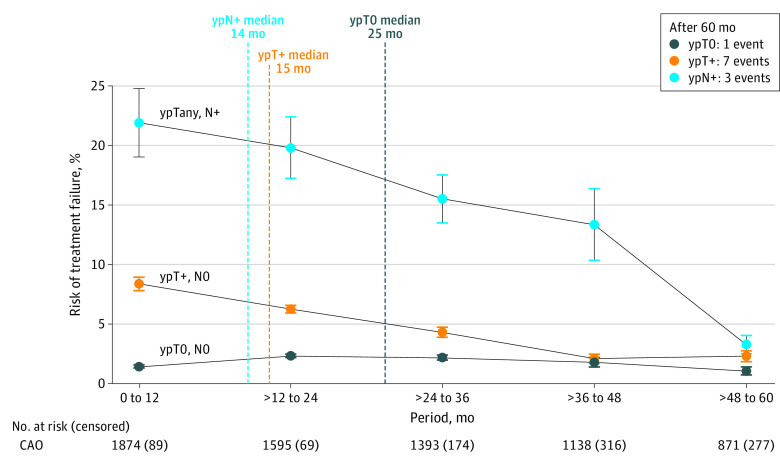
Risk of Treatment Failure Stratified by Pathologic Outcome Censored patients are patients who were lost during the specific follow-up period.

The risk of treatment failure was associated with the pathologic ypTNM stage. Within the first 12 months after randomization, 22% of patients with ypN+ experienced treatment failure vs only 1% of patients with a pathologic complete response (pCR) (Figure 3). Risk of treatment failure after pCR remained less than 3% in all evaluated time periods up to 5 years, whereas the median time to treatment failure was significantly increased to 25 months (IQR, 15-37 months) compared with 14 months (IQR, 7-27 months) after stage ypN+ (*P* = .04). Correlation analyses revealed a weakening correlation between DFS and OS from *R* = 0.93 in the CAO/ARO/AIO-94 trial to *R* = 0.77 in the CAO/ARO/AIO-12 trial (Spearman test, *P* < .001 for each trial; eFigure 3 in Supplement 1).

**Figure 4.**
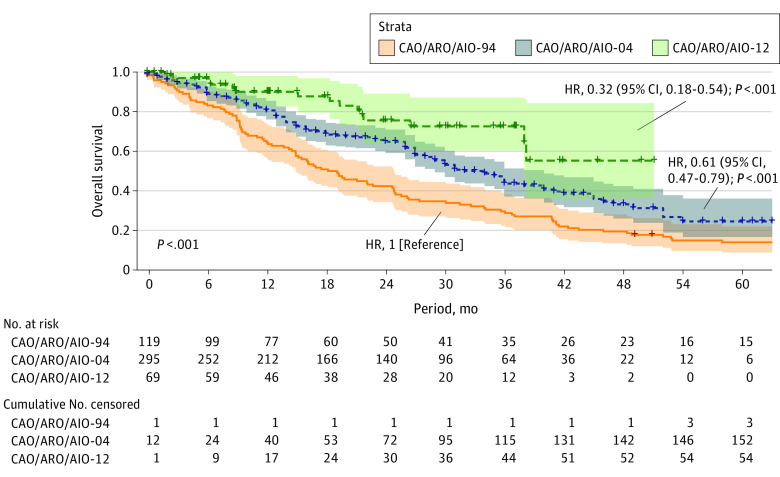
Overall Survival After Treatment Failure in the CAO/ARO/AIO-94, CAO/ARO/AIO-04, and CAO/ARO/AIO-12 Trials The log-rank test and a Cox proportional hazards regression model were used to assess statistical significance. The statistical tests were 2-sided. HR indicates hazard ratio.

With a median follow-up of 36 months (IQR, 24-51 months) for all patients, OS after treatment failure significantly improved in the CAO/ARO/AIO-04 trial (at 3 years, 44% [IQR, 37%-51%]; hazard ratio [HR], 0.61 [95% CI, 0.47-0.79]) and further in the CAO/ARO/AIO-12 trial (at 3 years, 73% [IQR, 60%-87%]; HR, 0.32 [95% CI, 0.18-0.54]) compared with the CAO/ARO/AIO-94 trial (at 3 years, 30% [IQR, 22%-39%]) (*P* < .001) (Figure 4). Median OS improved from 19 months (IQR, 15-25 months) in the CAO/ARO/AIO-94 trial to 34 months (IQR, 29-40 months) in the CAO/ARO/AIO-04 trial.

We also examined the association of tumor location (low, 0-5 cm; middle, >5-10 cm; high, >10 cm of the anal verge) and sex with treatment failure. Site of treatment failure did not differ by tumor location (eTable 1 in Supplement 1) or sex (eTable 2 in Supplement 1), nor was the median time to treatment failure different (15 months [IQR, 8-27 months] for male patients vs 13 months [7-24 months] for female patients; *P* = .08).

**Figure 5.**
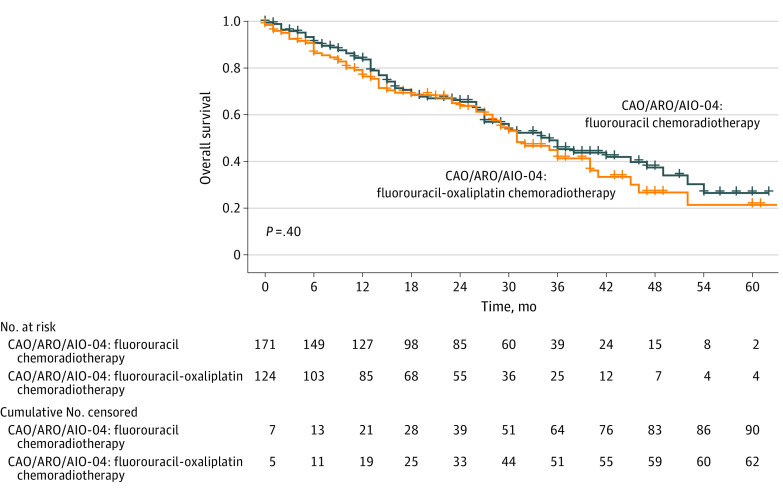
Overall Survival After Treatment Failure in the Fluorouracil vs the Fluorouracil-Oxaliplatin Groups of the CAO/ARO/AIO-04 Trials The log-rank test was used to assess statistical significance. The statistical tests were 2-sided.

Median OS after treatment failure tended to be higher among male (median, 31 months [IQR, 27-36 months]) than female patients (median, 26 months [IQR, 19-37 months]) (eFigure 4 in Supplement 1). The test of the moderation effect by sex indicates no significant difference in OS improvement between female and male patients (CAO/ARO/AIO-94 vs CAO/ARO/AIO-04 test of moderation effect by sex, *P* = .55; CAO/ARO/AIO-94 vs CAO/ARO/AIO-12 test of moderation effect by sex, *P* = .74), OS after treatment failure among male patients (eFigure 5 in Supplement 1), or OS after treatment failure among female patients (eFigure 6 in Supplement 1). We further investigated whether there was a difference in OS after treatment failure among patients treated with FU CRT or FU and oxaliplatin CRT in the CAO/ARO/AIO-04 trial. Of 625 patients treated with FU CRT, 171 (27%) experienced a treatment failure vs 124 of 607 patients (20.4%) treated with FU and oxaliplatin in the CAO/ARO/AIO-04 trial. The median time to treatment failure was 23 months (IQR, 11-35 months) after FU CRT vs 21 months (IQR, 9-31 months) after FU and oxaliplatin CRT (*t* test for difference, *P* = .27). The median OS after treatment failure was 35 months (IQR, 27-47 months) after FU CRT vs 31 months (IQR, 28-41 months) after FU and oxaliplatin CRT (HR, 1.2 [95% CI, 0.82-1.61]; *P* = .40) (Figure 5). Independent from the intensity of neoadjuvant and adjuvant treatment, both cohorts showed comparable improved OS after treatment failure compared with the CAO/ARO/AIO-94 trial (FU CRT: HR, 0.57 [95% CI, 0.43-0.77]; *P* < .001; FU and oxaliplatin CRT: HR, 0.66 [95% CI, 0.48-0.91]; *P* = .01). The test of the moderation effect by treatment indicates no significant differences (*P* = .53).

## Discussion

The time-dependent trend of OS among patients with rectal cancer after experiencing treatment failure after initial curative treatment has remained largely unexplored, to our knowledge. We examined the OS trends among patients with rectal cancer treated within 3 large randomized trials of the GRCSG during the past 3 decades. We provide evidence that OS after treatment failure has improved significantly in the more recent CAO/ARO/AIO-04 trial and, even more, in the CAO/ARO/AIO-12 trial compared with the older CAO/ARO/AIO-94 trial. This finding likely reflects the advancements in salvage treatment options, including systemic therapy with combination chemotherapy protocols, druggable molecular tumor targets (such as anti-angiogenesis [bevacizumab]), anti–epidermal growth factor receptor (cetuximab or panitumumab), and immunotherapy (pembrolizumab), as well as improvements in surgery and radiotherapy (eg, local stereotactic ablative radiotherapy for oligometastatic treatment) as part of the multidisciplinary approach for patients with rectal cancer who experienced treatment failure after initial curative therapy during the past decades.^12,13,14,15,16,17^ Furthermore, a more successful salvage treatment also provides a possible explanation for the lack of correlation between DFS and OS in the more recent trials of the GRCSG.^6,18^ Overall survival after treatment failure improved to a comparable extent among both male and female patients. In line with previous analyses regarding the association of sex with toxic effects, adherence, and outcomes among patients with rectal cancer,^19^ we did not find a significant difference in outcomes between male and female patients in the present analysis. The median OS after treatment failure tended to be higher among male (median, 31 months [IQR, 27-36 months]) than female (median, 26 months [IQR, 19-37 months]) patients. This finding is in contrast to the increased survival for female patients with colorectal cancer reported in the meta-analysis by Yang et al.^20^ Further analysis on the potential reasons for these conflicting results is needed.

The RAPIDO and the PRODIGE23 randomized phase 3 trials showed improved pCR and DFS but initially failed to demonstrate improved OS after TNT compared with CRT, surgery, and (optional) adjuvant chemotherapy.^1,2^ At the 2023 American Society of Clinical Oncology Annual Meeting, improved OS was reported for the experimental group of the PRODIGE23 trial (OS at 7 years: standard treatment group, 76.1% vs experimental group, 81.9%).^21^ A recent post hoc analysis of the RAPIDO trial confirmed DM as the main site of treatment failure (lung, 13%; liver, 9%) in the TNT era, while reporting that TNT reduced the occurrence of liver, but not lung, metastases compared with CRT.^4^ Primary salvage treatment approaches were surgery (46%), radiotherapy (14%), and chemotherapy (50%). The timing of metastases was comparable between CRT and TNT, but patients who developed DM after TNT had a significantly shorter OS after treatment failure (median, 2.6 vs 3.2 years), in line with a proposed possible ATRESS (Neoadjuvant Therapy-Related Shortening of Survival) phenomenon in rectal cancer. ATRESS implies a reduction in post–treatment failure survival after intensified neoadjuvant treatment (eg, by induction of chemoresistance in remaining surviving tumor clones or by limiting the use of chemotherapy [eg, oxaliplatin] for patients who experience treatment failure).^4,22^ By contrast, in our study, OS after treatment failure did not significantly change after initial intensification of treatment in the CAO/ARO/AIO-04 trial (FU vs FU and oxaliplatin). In the PRODIGE23 trial, the median OS after metastatic disease in the TNT group was even longer than in the CRT group (44.4 months vs 39.4 months; *P* = .06).^23^ The association of intensified neoadjuvant treatment with OS after treatment failure needs to be further investigated.

### Limitations

This study has some limitations. The reason for improved OS after treatment failure over time cannot be stated with certainty based on our data because we cannot provide detailed information on individual salvage strategies. Nevertheless, we tried to address potential biases. First, time to treatment failure was not significantly different in the 3 trials; hence, the notion that modern surveillance programs could have led to earlier detection of treatment failure and an earlier start of salvage therapy is not valid. Nevertheless, our findings that the relative risk of experiencing an LR as treatment failure increased in the third and fourth year of follow-up could have implications in the context of surveillance programs. Currently, the German guideline recommends proctoscopy and/or colonoscopy after surgery only up to 24 months, but not thereafter, which might need adaptation in the future.^24^ Second, the median age of patients, which could have had an association with OS, was comparable among the trials (63, 63, and 60 years in the CAO/ARO/AIO-94, -04, and -12 trials, respectively). Third, different adherence to surveillance programs may be associated with detection of DM and OS. Only 3 patients were censored in the CAO/ARO/AIO-94 trial throughout the follow-up, compared with 152 in the CAO/ARO/AIO-04 trial and 54 in the CAO/ARO/AIO-12 trial. Different proportions of censoring events should be considered when interpreting our results.

## Conclusions

In this cohort study, we demonstrated a time-dependent improvement in OS after treatment failure among patients with locally advanced rectal cancer treated within 3 consecutively recruiting randomized phase 2 or 3 trials of the GRCSG,^5,6,7,8,9,10^ which likely reflects advancements in individual salvage treatment options during the past decades.

## Notes

Supplement 1. **eFigure 1.** Study Flow Diagram of the Present Post Hoc Analysis **eFigure 2.** Risk of Treatment Failure Within Certain Follow-up Periods, Depending on Trial **eFigure 3.** Correlation Between DFS in Months and OS in Months in the CAO/ARO/AIO-94 Trial (A), in the CAO/ARO/AIO-04 Trial (B) and the CAO/ARO/AIO-12 Trial (C) Correlation Were Analyzed Using the Spearman Test **eTable 1.** Association of Tumor Localization and Cause of Treatment Failure **eTable 2.** Association of Sex and Cause of Treatment Failure **eFigure 4.** Overall Survival After Treatment Failure in Male and Female Patients **eFigure 5.** Overall Survival After Treatment Failure in Male Patients **eFigure 6.** Overall Survival After Treatment Failure in Female Patients

Supplement 2.
Data Sharing Statement
