# Supplementary material for: Overall Survival After Treatment Failure Among Patients With Rectal Cancer
Source: JAMA Netw Open. 2023 Oct 30;6(10):e2340256. doi: 10.1001/jamanetworkopen.2023.40256 (PMC10616722; doi:10.1001/jamanetworkopen.2023.40256)
Supplement: Supplement 1. — eFigure 1. Study Flow Diagram of the Present Post Hoc Analysis eFigure 2. Risk of Treatment Failure Within Certain Follow-up Periods, Depending on Trial eFigure 3. Correlation Between DFS in Months and OS in Months in the CAO/ARO/AIO-94 Trial (A), in the CAO/ARO/AIO-04 Trial (B) and the CAO/ARO/AIO-12 Trial (C) Correlation Were Analyzed Using the Spearman Test eTable 1. Association of Tumor Localization and Cause of Treatment Failure eTable 2. Association of Sex and Cause of Treatment Failure eFigure 4. Overall Survival After Treatment Failure in Male and Female Patients eFigure 5. Overall Survival After Treatment Failure in Male Patients eFigure 6. Overall Survival After Treatment Failure in Female Patients [file jamanetwopen-e2340256-s001.pdf]

Supplementary Online Content

Diefenhardt M, Martin D, Fleischmann M, et al. Overall survival after treatment failure among patients with rectal cancer. *JAMA Netw Open.* 2023;6(10):e2340256. doi:10.1001/jamanetworkopen.2023.40256

eFigure 1. Study Flow Diagram of the Present Post Hoc Analysis

eFigure 2. Risk of Treatment Failure Within Certain Follow-up Periods, Depending on Trial

eFigure 3. Correlation Between DFS in Months and OS in Months in the CAO/ARO/AIO-94 Trial (A), in the CAO/ARO/AIO-04 Trial (B) and the CAO/ARO/AIO-12 Trial (C) Correlation Were Analyzed Using the Spearman Test

eTable 1. Association of Tumor Localization and Cause of Treatment Failure

eTable 2. Association of Sex and Cause of Treatment Failure

eFigure 4. Overall Survival After Treatment Failure in Male and Female Patients

eFigure 5. Overall Survival After Treatment Failure in Male Patients

eFigure 6. Overall Survival After Treatment Failure in Female Patients

This supplementary material has been provided by the authors to give readers additional information about their work.

eFigure 1. Study Flow Diagram of the Present Post Hoc Analysis

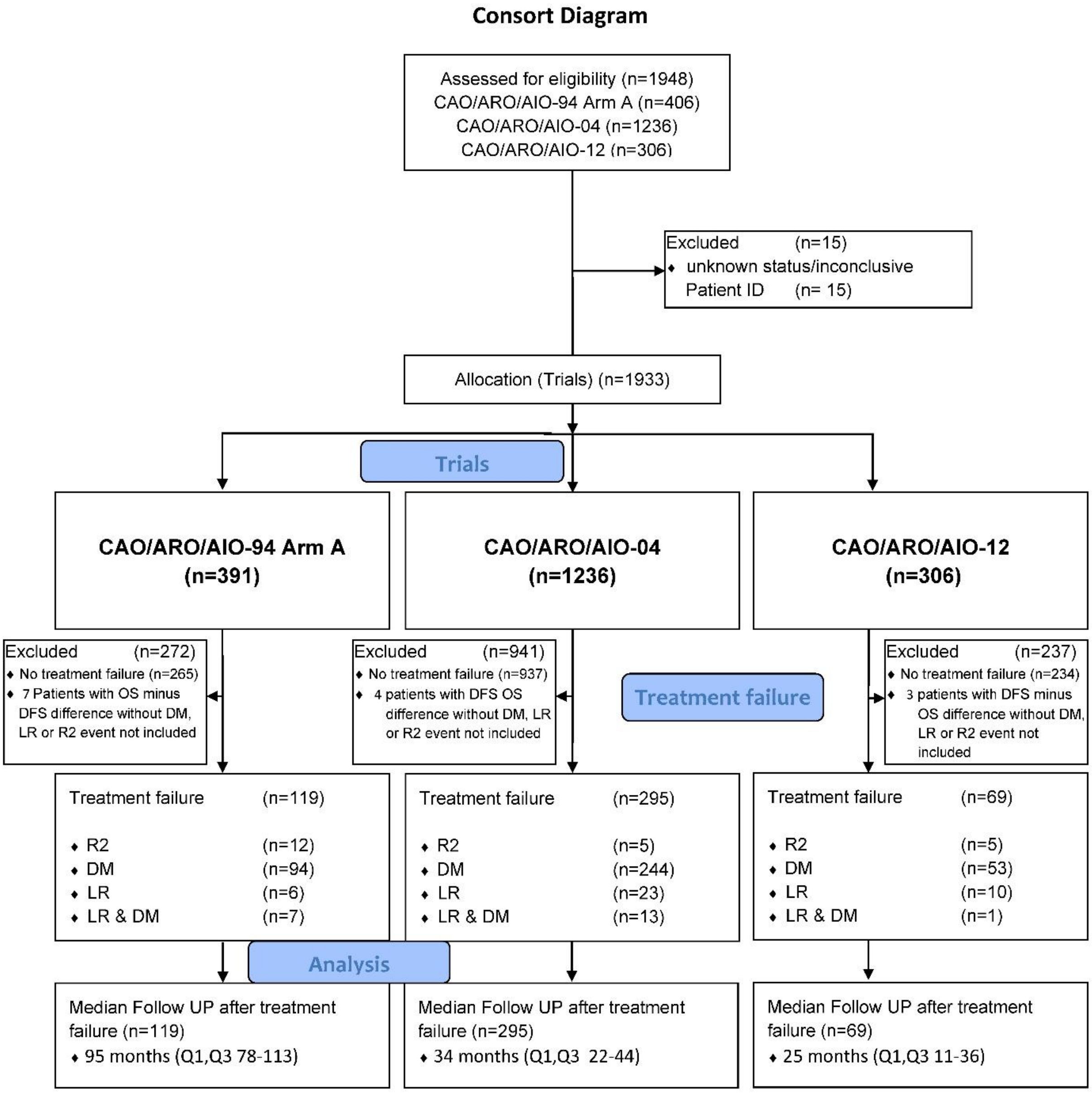

* Sauer R et al.; Preoperative versus postoperative chemoradiotherapy for locally advanced rectal cancer: results of the German CAO/ARO/AIO-94 randomized phase III trial after a median follow-up of 11 years. J Clin Oncol. 2012

** Rödel C et al.; German Rectal Cancer Study Group. Oxaliplatin added to fluorouracil-based preoperative chemoradiotherapy and postoperative chemotherapy of locally advanced rectal cancer (the German CAO/ARO/AIO-04 study): final results of the multicentre, open-label, randomised, phase 3 trial. Lancet Oncol. 2015

*** Fokas E et al.; Chemoradiotherapy Plus Induction or Consolidation Chemotherapy as Total Neoadjuvant Therapy for Patients With Locally Advanced Rectal Cancer: Long-term Results of the CAO/ARO/AIO-12 Randomized Clinical Trial. JAMA Oncol. 2022

© 2023 Diefenhardt M et al. *JAMA Network Open.*

eFigure 2. Risk of Treatment Failure Within Certain Follow-up Periods, Depending on Trial

A: Risk of treatment failure in complete cohort
B: Risk of treatment failure in the CAO/ARO/AIO-94 trial
C: Risk of treatment failure in the CAO/ARO/AIO-04 trial
D: Risk of treatment failure in the CAO/ARO/AIO-12 trial
Censored patients are patients who were lost during the specific follow-up period.

© 2023 Diefenhardt M et al. *JAMA Network Open.*

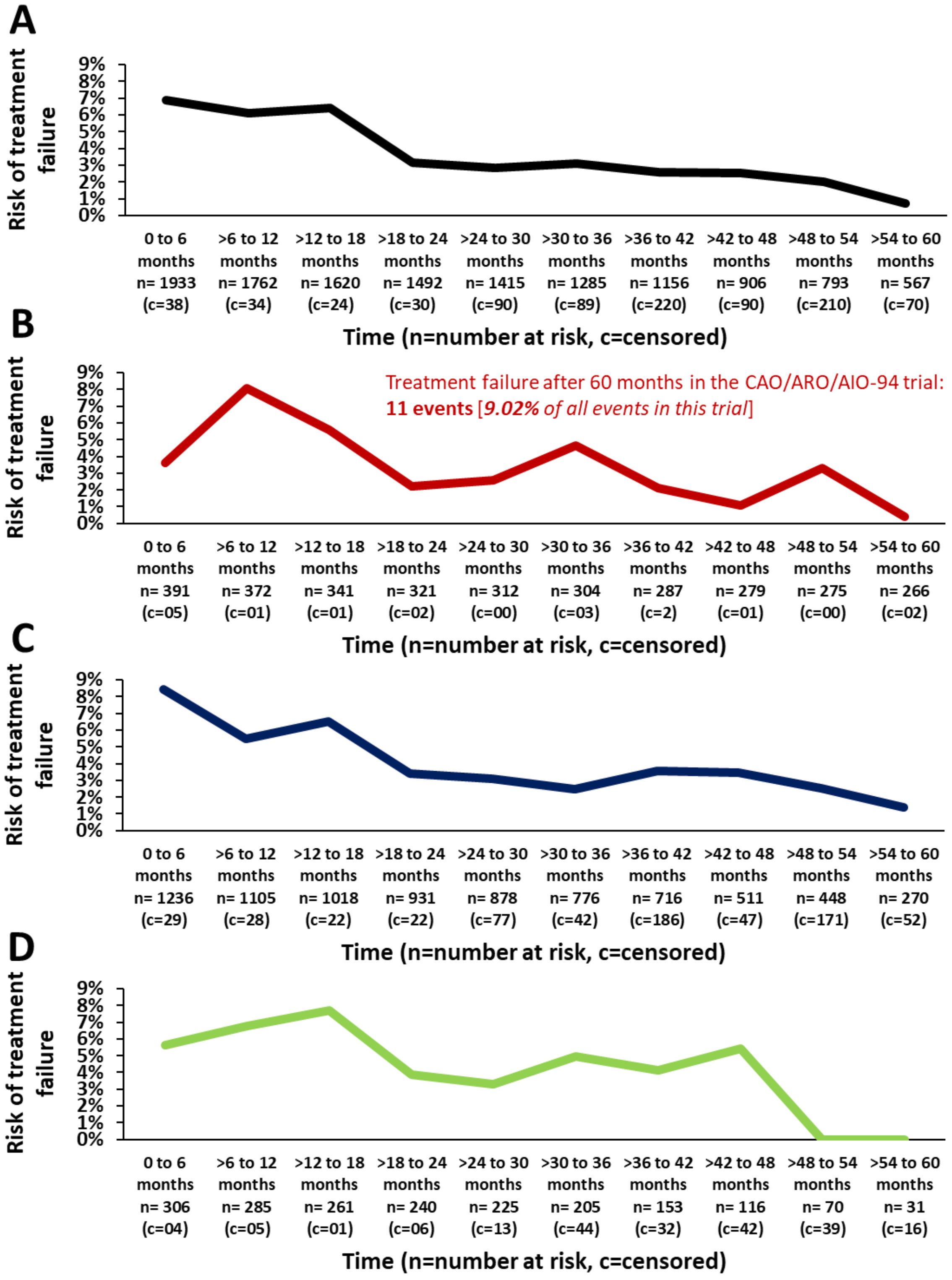

© 2023 Diefenhardt M et al. *JAMA Network Open.*

eFigure 3. Correlation Between DFS in Months and OS in Months in the CAO/ARO/AIO-94 Trial (A), in the CAO/ARO/AIO-04 Trial (B) and the CAO/ARO/AIO-12 Trial (C) Correlation Were Analyzed Using the Spearman Test.

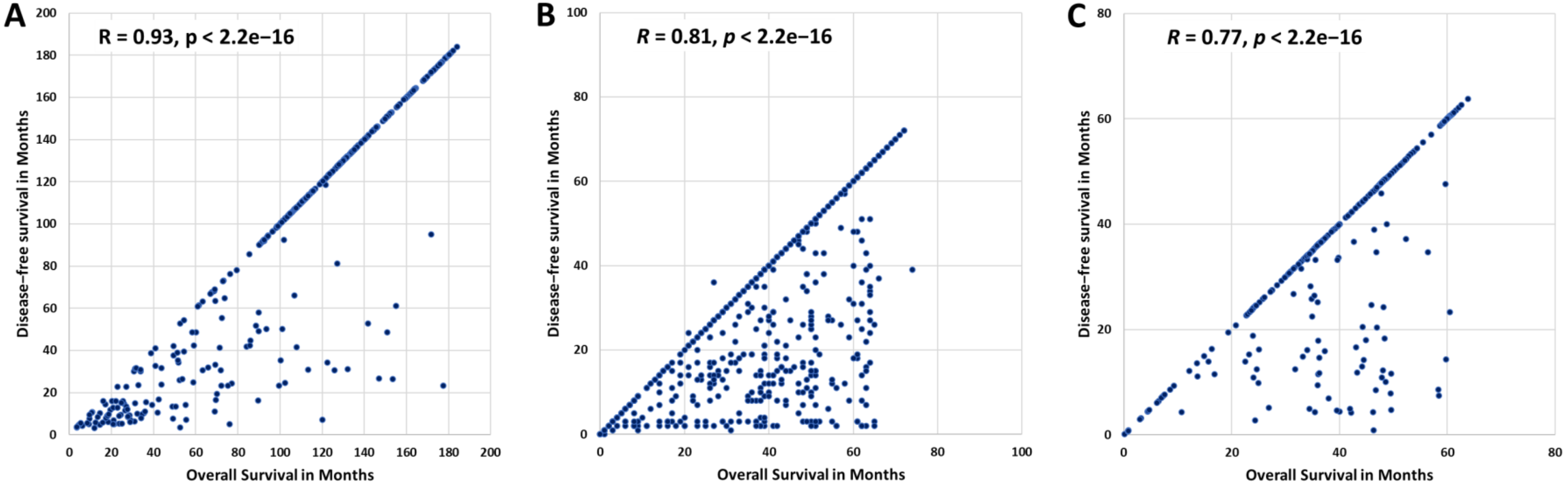

© 2023 Diefenhardt M et al. *JAMA Network Open.*

eTable 1. Association of Tumor Localization and Cause of Treatment Failure

Treatment failure by tumor localisation	Low<br>< 0 cm to 5 cm of the anal verge	Intermediate<br>> 5 cm to 10 cm of the anal verge	High<br>> 10 cm of the anal verge
	n= 222	n= 218	n= 43
local recurrence and distant metastasis	12 (5.4%)	7 (3.2%)	2 (4.7%)
distant metastasis	176 (79.3%)	179 (82.1%)	36 (83.7%)
local recurrence	19 (8.6%)	18 (8.3%)	2 (4.7%)
R2 resection	15 (6.8%)	14 (6.4%)	3 (7.0%)

© 2023 Diefenhardt M et al. *JAMA Network Open*.

eTable 2. Association of Sex and Cause of Treatment Failure

Treatment failure by sex	male patients	female patients
	n=343	n=140
local recurrence and distant metastasis	17 (5.0%)	4 (2.9%)
distant metastasis	274 (79.9%)	117 (83.6%)
local recurrence	28 (8.2%)	11 (7.9%)
R2 resection	24 (7.0%)	8 (5.7%)

© 2023 Diefenhardt M et al. *JAMA Network Open.*

eFigure 4. Overall Survival After Treatment Failure in Male and Female Patients

The log-rank test and a Cox regression model were used to assess statistical significance. The statistical tests were two-sided.

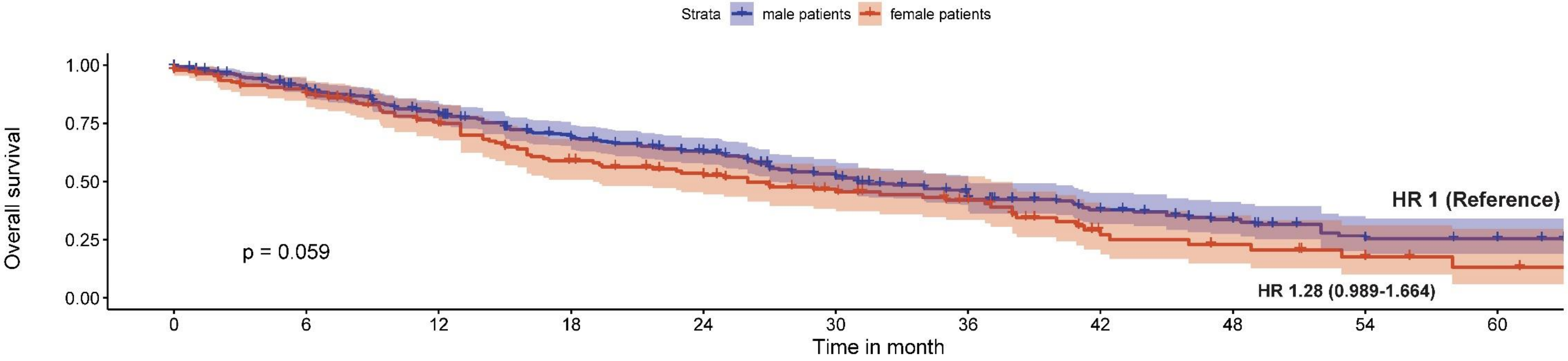

Number at risk

	0	6	12	18	24	30	36	42	48	54	60
male patients	343	290	241	195	160	115	80	51	37	22	18
female patients	140	120	94	69	58	42	31	14	10	6	3

Time in month

Cumulative number of censoring

	0	6	12	18	24	30	36	42	48	54	60
male patients	11	26	42	59	78	99	119	134	145	150	155
female patients	3	8	16	19	25	33	41	49	50	53	54

Time in month

© 2023 Diefenhardt M et al. *JAMA Network Open.*

eFigure 5. Overall Survival After Treatment Failure in Male Patients

The log-rank test and a Cox regression model were used to assess statistical significance. The statistical tests were two-sided.

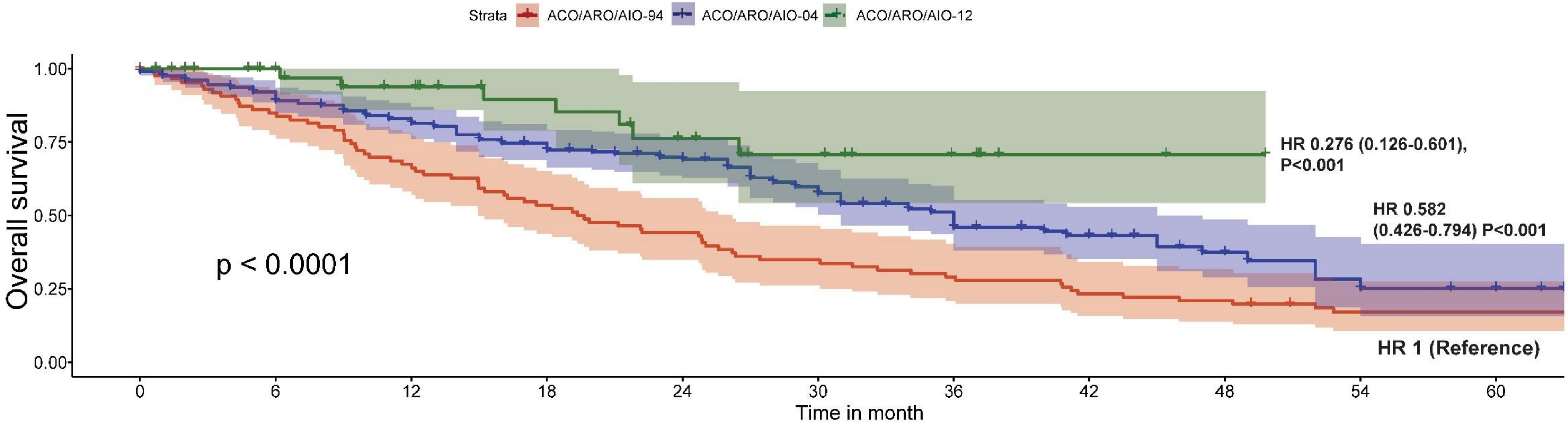

Number at risk

	0	6	12	18	24	30	36	42	48	54	60
CAO/ARO/AIO-94	87	73	58	46	38	30	25	20	18	13	13
CAO/ARO/AIO-04	215	183	156	128	107	75	49	29	18	9	5
CAO/ARO/AIO-12	41	34	27	21	15	10	6	2	1	0	0

Time in month

Cumulative number of censoring

	0	6	12	18	24	30	36	42	48	54	60
CAO/ARO/AIO-94	1	1	1	1	1	1	1	1	1	3	3
CAO/ARO/AIO-04	10	17	29	41	57	74	90	101	111	113	118
CAO/ARO/AIO-12	0	8	12	17	20	24	28	32	33	34	34

Time in month

© 2023 Diefenhardt M et al. *JAMA Network Open.*

eFigure 6. Overall Survival After Treatment Failure in Female Patients

The log-rank test and a Cox regression model were used to assess statistical significance. The statistical tests were two-sided.

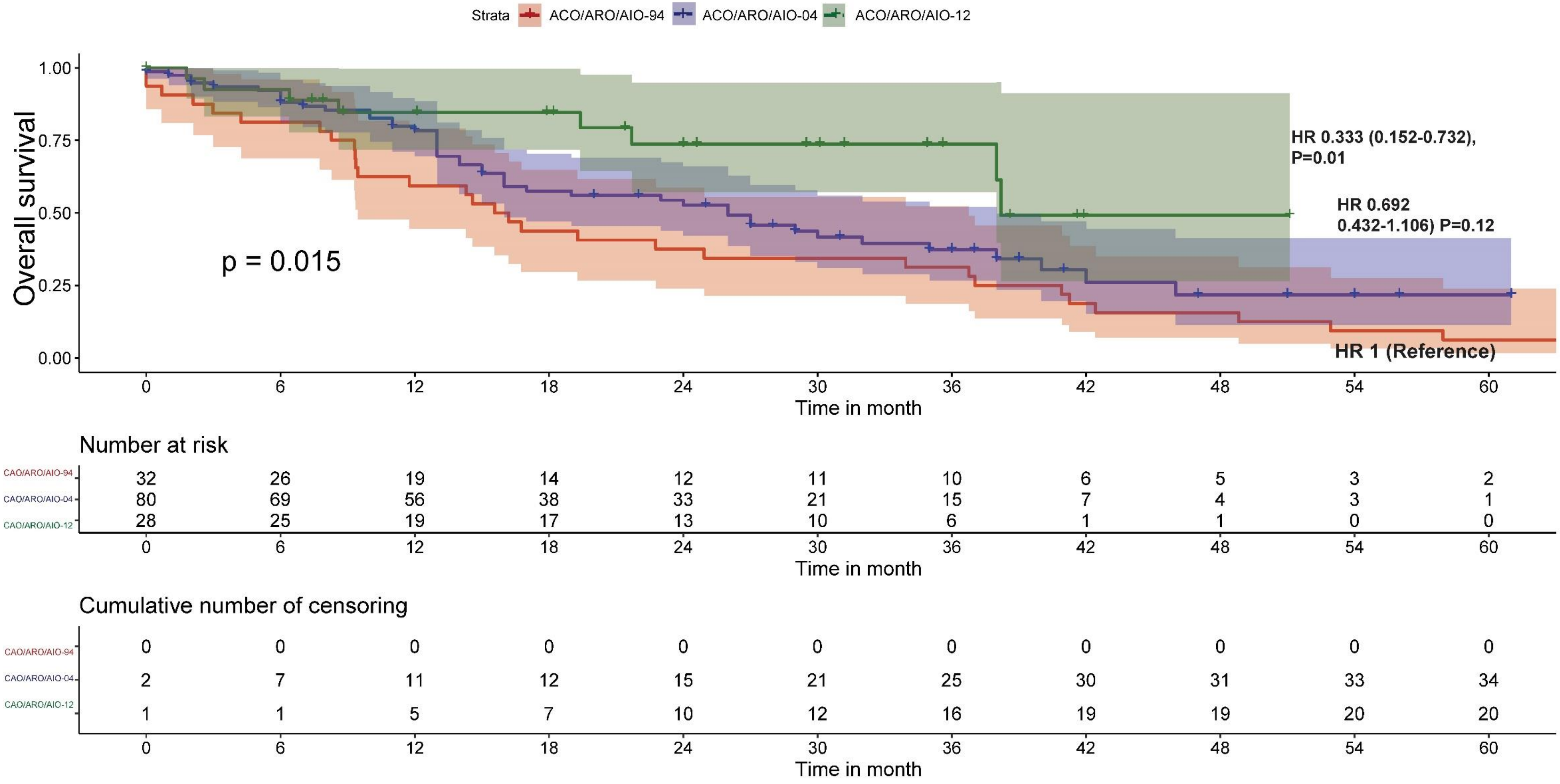

© 2023 Diefenhardt M et al. *JAMA Network Open.*
