# Supplementary material for: Overall Survival After Treatment Failure Among Patients With Rectal Cancer
Source: JAMA Netw Open. 2023 Oct 30;6(10):e2340256. doi: 10.1001/jamanetworkopen.2023.40256 (PMC10616722; doi:10.1001/jamanetworkopen.2023.40256)
Supplement: Supplement 2. — Data Sharing Statement [file jamanetwopen-e2340256-s002.pdf]

Data Sharing Statement

Diefenhardt. Overall Survival After Treatment Failure Among Patients With Rectal Cancer. *JAMA Netw Open*. Published October 30, 2023. doi:10.1001/jamanetworkopen.2023.40256

Data
Data available: No

Additional Information
Explanation for why data not available: Patients enrolled in the trials did not provide informed consent for the data to be shared publicly. Therefore, data from the trials cannot be shared publicly.
